# A Systematic Review of Vertigo: Negligence in Pregnancy

**DOI:** 10.7759/cureus.29814

**Published:** 2022-10-01

**Authors:** Vaishnavi Toshniwal, Aman Agrawal, Tejas Toshniwal, Saket Toshniwal, Sankalp Khanke, Sanket Bakshi, Neema Acharya

**Affiliations:** 1 Department of Medicine, Jawaharlal Nehru Medical College, Datta Meghe Institute of Medical Sciences (Deemed University), Wardha, IND; 2 Department of Medicine, Jawaharlal Nehru Medical College, Datta Meghe Institute of Medical Sciences (Deemed to be University), Wardha, IND; 3 Department of Obstetrics and Gynaecology, Jawaharlal Nehru Medical College, Datta Meghe Institute of Medical Sciences (Deemed University), Wardha, IND

**Keywords:** vestibulocochlear nerve diseases, pregnancy, vestibular disease, dizziness, vertigo

## Abstract

From conception to childbirth, there are many physical, hormonal, and psychological changes that a woman undergoes during pregnancy. During this time, balance is also affected, resulting in symptoms like vertigo and unsteadiness. These symptoms can lead to physical impairment and disability and can develop at any time. Vertigo in pregnancy has not been extensively written about. The subject of a narrative review is vertigo in pregnant patients. In pregnant women, hormonal alterations in the peripheral tissues and inner ear organs may contribute to vertigo. Meniere's disease, mild convulsive positional dizziness, and oculomotor migraines are all commonly exacerbated by pregnancy. Between the second and third trimesters of pregnancy, specific modifications to proprioception and hearing are also detected during physical examination. Patients who are pregnant typically experience these symptoms throughout this time. Some vertigo conditions can worsen during pregnancy, while others can appear at any time. Understanding audio-vestibular symptoms' pathological and clinical relationship during pregnancy requires more study.

## Introduction and background

Pregnancy involves nine months of physiological change in numerous organs, which include hormonal, cardiovascular, and psychological alterations. Progesterone, estrogen, placental lactogen, human chorionic gonadotropin, and relaxin regulate many of these changes, which affect the anatomical structure and functionality of the gastrointestinal, respiratory, cutaneous, cardiovascular, musculoskeletal, and audio-vestibular systems [1].

Pregnancy can bring about the onset of or exacerbate several audiovestibular system disorders, including autophony, hearing loss, tinnitus, otosclerosis, vertigo, and facial paralysis [2]. It could be described by how estrogen and progesterone affect specific balance and hearing-related structures like the stria vascularis, cochlea, and spiral ligament, which results in osmolar and chemical changes in the endolymphatic fluids. This crucial substance has been involved in the governance of the inner ear [3]. But the hormone overflow impacts more than just these structures [4]. Gait disturbances and falls may result from impaired proprioception and cognition brought on by vascular and electrolytic activity on peripheral receptors [5].

However, we see that these signs are usually taken less seriously during the childbearing period. A few studies have suggested that vertigo during pregnancy is expressed in those with a history of Meniere's disease, while others have suggested that the beginning of vertigo is related to vestibular neuritis [2]. The occurrence of shakiness, syncope, and dysautonomia during this time, which pregnant women commonly experience, is another challenge. This makes it more challenging for clinicians and otolaryngologists to diagnose and treat vertigo. This article sought to examine the clinical signs, prevalence, and common forms of dizziness experienced by pregnant patients.

Methodology

Scopus, PubMed, and EBSCO Information Services were searched for articles published in Spanish and English between February 1999 and November 2022. Medical subject heading (MeSH) terms like " vertigo", "dizziness", "vestibular disease", "pregnancy", and "vestibulocochlear nerve diseases" have been used in this literature search. Articles describing vertigo during pregnancy were eligible for inclusion if they included the following study types: cross-sectional studies, case reports, systemic reviews, retrospective chart reviews, and case-control-cohorts. Also, reports of Meniere's disease, benign paroxysmal position vertigo (BPPV), vestibular-neuritis, as well as different peripheral vertigo presentations in the childbearing period, have been considered. The following were not included: scope reviews, editorials, letters-to-the-editor, narrative reviews, and abstracts. Some of the articles that did not address vertigo during pregnancy, the onset of the condition earlier in pregnancy, and the early period have been disregarded. The report's findings on dizziness in the pregnancy period linked to central nervous system disorders, arteriovenous malformations, and inner ear anomalies were also ignored. There were 105 articles found in total, 25 of which were used for this review. The authors double-checked each of the articles they chose.

**Figure 1:**
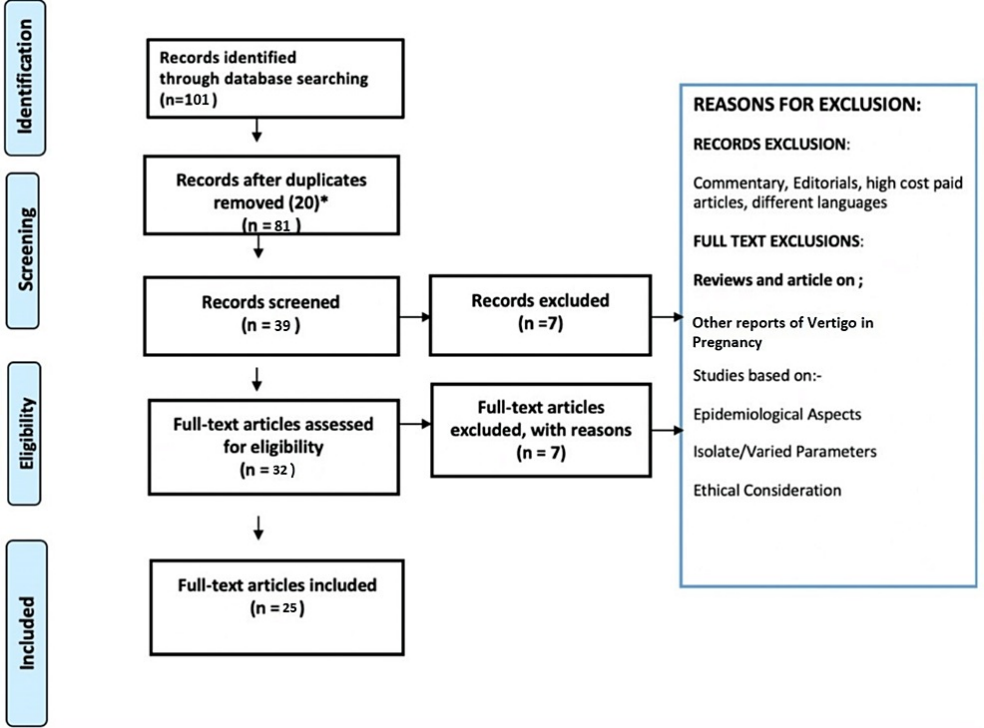
A flowchart showing the methodology and study selection using the PRISMA method.

In this literature review, the Preferred Reporting Items for Systematic Reviews and Meta-Analyses (PRISMA) method was used. We included certain observational studies, case reports, reviews, and various original scientific research papers. We included studies that depicted a correlation between vertigo and pregnancy. Only the most recent articles were considered for this review. A flowchart showing the methodology and study selection is presented in Figure 1.

## Review

The pathology and clinical manifestations of vertigo during pregnancy 

The illusion of movement is what is referred to as vertigo [6-8]. This subjective perception may differ between a patient's internal and external environments [8]. At least 80% of people worldwide have experienced vertigo at some point in their life, making it one of the most common illnesses seen during emergency rooms and office appointments [8,9]. Annual incidence climbs by up to 30% and is approximately 7% globally [9]. Around the world, females are typically more afflicted than males (1:2.7) [9]. In up to 70% of instances, this incapacitating and disabling symptom is linked to impaired inner ear function [9]. However, there are other elements involved with the onset of vertigo [8], in addition to disruptions of the inner ear's architecture and function. The pathophysiology and equilibrium of vertigo are also influenced by vision, proprioception, and central nervous integration. [8]. Also, because of the connected vestibulomotor system, spinal cord, and chemoreceptor trigger zone-vomiting center, symptoms like nausea, gait imbalance, vomiting, falls, and unsteadiness can appear. [9-13].

Vertigo is one of the most common symptoms that pregnant women report to their primary care doctors [14]. For every 100,000 new instances reported annually in the U.S., 32 pregnant women see a primary care doctor with vertigo [14,15]. It is rare for primary care doctors, gynecologists, and E.N.T. specialists to treat these patients for this common complaint; yet, because it is so widespread and linked to metabolic, orthostatic, and functional abnormalities, it is rarely researched [15]. It may be explained by a patient's lifetime changes in metabolism, hormonal fluctuations, and hereditary predisposition [11,12]. This symptom may be expected during the fertile decade, particularly during pregnancy, yet it is still underdiagnosed. During this time, steroidal sex hormones like estrogen are produced more frequently, altering the functions of the adrenal gland and placenta [11,12]. Other than this, the central nervous and vestibular systems may also be impacted [13].

The inner ear is responsible for two critical processes: hearing and balance. Hormones like estrogen in the endolymphatic fluid may have an impact on stria vascularis, cochlea, spiral ligament, and spiral ganglion neurons, as well as on the neuron's afferent termination. Additionally, a few enzyme receptors on Na+/K+ (sodium/potassium) channels in the cochlea and labyrinth of the membrane are affected by estrogen, which results in the development of otological signs like vertigo and dizziness in pregnant women [16-19]. Other events related to the inner ear include fluid retention in perilymph and endolymph, as well as hypercoagulability in the auditory arteries.

The labyrinthine artery and its branches are more prone to vascular occlusion, which is harmful [16-19]. Increases in several factors of coagulation (VII, VIII, IX, X, XII) and fibrinogen with the reduction in factor XI are mainly associated with occlusions, which are often seen during the first trimester of pregnancy and stabilize in the second or third trimester [19]. Thus, pregnancy is considered a hypercoagulable state with increased activation of the blood clotting and fibrinolysis systems that would increase plasma viscosities and erythrocytic aggregations and reduce deformities. Therefore, pregnant women have a higher chance of developing thromboembolism in the labyrinthine arteries and labyrinthine micro-circulation occlusion [19].

Estrogen and progesterone are sex steroid hormones that are produced and excreted at much higher rates during pregnancy [19]. Circulating levels of progesterone have been as high as 20 times more significant in the third trimester compared to normal, and estradiol levels are 30 to 40 times higher as compared to what they are during menstruation [20]. The increased sodium and water retention brought on by these hormone changes have resulted in electrolytic imbalance and an increase in the amount of extracellular fluid [20]. Other changes in estrogen during the childbearing period are related to the function of the brain, and throughout different gestational weeks, low estrogen levels are linked to problems with spatial orientation [7]. Hormonal changes during pregnancy can cause auditory fullness and loss of hearing at a lower frequency, which can be corrected after delivery [16]. According to Naftalin et al., symptoms such as vertigo and aural fullness first appear in early pregnancy, which go into remission before exacerbating during the second trimester and lactation [17].

Instability and imbalance were more common during the second trimester than they were in the first [10]. Instability and a propensity to fall occur more often in the third trimester [10]. The same study suggests that dizziness symptoms in the first trimester are caused by a vestibular change caused by a hormonal change and that dizziness symptoms in the subsequent trimesters are caused by labyrinthine habituation [10]. There was no link between weight gain and balance, and these symptoms persisted throughout the postpartum period. This suggests that postural instability in this population is more closely related to hormones, ligaments, and joint changes than weight gain. Thus, there would be a greater decrease in balance in pregnant women as compared to non-pregnant women.

Vertigo can occur in the childbearing stage. Patients who have previously been diagnosed with vestibular diseases like Meniere's disease or vestibular migraine may experience worsening vertigo [5]. Up to 57 percent of patients with Meniere's disease and up to 50 percent of patients with vestibular migraine experience flare-ups in the third trimester, respectively [5]. In addition, benign paroxysmal positional vertigo (BPPV ) frequently occurs during pregnancy. The following describes these prevalent disorders.

Meniere’s disease

Meniere's disease is an episodic form of vertigo that is linked to problems with the regulation of inner ear endolymphatic fluid, leading to obstruction and an increase in the endolymphatic fluid that can be clinically manifested as loss of hearing, tinnitus, fullness, and vertigo [21]. During pregnancy, a highly turbulent osmotic gradient known as hydrops affects the endolymphatic sac, saccule, cochlea, and semicircular canals, which is a result of a decrease in the osmolality of systemic and local fluids to the ear during pregnancy [7].

Meniere's disease is frequently linked to unexpected hearing loss or dizziness during the second and third trimesters in pregnant individuals. Meniere's disease frequently develops during pregnancy and may go away after delivery for many patients. Meniere's disease may worsen in patients who have already been diagnosed with it in the second and third trimesters [18-21]. During the first trimester, some patients are under control, and their symptoms improve [21]. The Barany Society Criteria are used to confirm the diagnosis of Meniere's disease. At least four of the following conditions must be present: (1) between 20 minutes and 12 hours, there must be two or more episodes of spontaneous vertigo; (2) audiometry-confirmed low-and mid-frequency sensorineural hearing loss in one ear, with the affected ear identified at least once before, during, or after one of the vertigo episodes; (3) changing auditory symptoms in the affected ear, such as hearing loss, tinnitus, or fullness; and (4) no other vestibular diagnosis can explain the symptoms better [21].

Salt and caffeine restrictions are advised for the treatment of Meniere's disease [18]. Although betahistine is rarely used during pregnancy, it must occasionally be administered with prudence [18]. Antipsychotics like prochlorperazine should be used cautiously in the third trimester, as these are linked to extrapyramidal effects in the newborn baby [18].

Benign paroxysmal positional vertigo ( BPPV)

BPPV is characterized as an episodic form of vertigo that is triggered by certain changes in head position, like spinning. This is the most prevalent peripheral vestibular disease in which women are affected more commonly than males [22]. When all three semicircular canals are compared, posterior involvement (approximately 85 to 95 percent) is more common than lateral (horizontal semicircular canal) involvement [22]. Most BPPV patients involve the posterior canal [22].

BPPV may worsen during pregnancy as a result of left-sided sleeping (as it has been advised to do to lessen vena cava compression during pregnancy), prolonged bed rest, disorders of vitamin D deficiency, and calcium metabolism during the second trimester linked to increasing reabsorption of calcium in many systems, as in the kidneys and bones, and higher metabolic demands of the fetus [23].

Vestibular migraine

Episodic vertigo, coupled with a variety of symptoms, including visual auras, photophobia, phonophobia, pulsating unilateral headache, and light sensitivity, is known as vestibular migraine [24,25]. Most people have had a migraine diagnosis in the past [24]. Vestibular migraine affects between 1.1 and 3.2 percent of the population and can occur at any age. Females outnumber males by a factor of 1.5:5 [24]. Numerous hypotheses have been made, all of which are based on the pathophysiology of migraine. These hypotheses include neurochemical, genetic, and inflammatory processes, even though the origin of vestibular migraine is still unknown [24].

Vestibular migraines can occur in up to 40% of pregnant women, and the time that these individuals feel vertigo can range from minutes to hours. Compared to non-pregnant individuals, these patients may regularly report tinnitus and osmophobia in both ears, in addition to the usual vestibular migraine symptoms [24].

In patients with long-term vestibular migraine, saccadic pursuits or persistent positional nystagmus are typically observed [25]. Other findings in 10-20% of pregnant women include greater contralateral predominance, lower unilateral caloric responses, and increased unilateral vestibular deficits [25]. 

## Conclusions

Vertigo is a common symptom in pregnant individuals, and different varieties of this symptom might signify various vestibular illnesses like Meniere’s disease, vestibular migraine, and benign paroxysmal positional vertigo. From the start of pregnancy till childbirth, vascular and hormonal alterations are part of the etiology of vertigo. More clinical research is required to comprehend how vertigo impacts each trimester and how it could impact fetal development.
